# Examining Interpregnancy Weight Change Across a Birthing Population in Aotearoa, New Zealand

**DOI:** 10.1111/ajo.70037

**Published:** 2025-05-23

**Authors:** Emma Le Lievre, Per Kempe, Robin Cronin, Alice Hyun Min Kim, Rosemary Hall

**Affiliations:** ^1^ Otago University (Wellington) Wellington New Zealand; ^2^ Healthy Women, Children and Youth MidCentral Palmerston North New Zealand; ^3^ Women's Health Division Counties Manukau Health Auckland New Zealand; ^4^ Department of Obstetrics and Gynaecology, Faculty of Medical and Health Sciences The University of Auckland Auckland New Zealand; ^5^ Biostatistics Group, Dean's Department Otago University (Wellington) Wellington New Zealand; ^6^ Department of Medicine Otago University (Wellington) Wellington New Zealand

**Keywords:** body mass index, change, inter‐pregnancy, weight

## Abstract

**Introduction:**

Inter‐pregnancy weight change (IPWC) has been linked to adverse outcomes in subsequent pregnancies. No studies have examined IPWC in Aotearoa New Zealand (AoNZ). This study aims to 1. Examine the distribution of IPWC in a birthing population in AoNZ and 2. Investigate IPWC across priority populations identified by the Perinatal and Maternal Mortality Review Committee (PMMRC).

**Methods:**

This retrospective cohort study in AoNZ, included people who birthed their first two singleton pregnancies between 2016 and 2021. IPWC was calculated as the difference in pre‐pregnancy body mass index (BMI) and divided into four categories: 1. Interpregnancy weight loss (IPWL) (BMI reduction of > 1 kg/m^2^) no change in weight (NC) (BMI change −1–0.9 kg/m^2^); moderate interpregnancy weight gain: (BMI increase 1–3 kg/m^2^) and increased IPWG (BMI increase > 3 kg/m^2^).

**Results:**

The study cohort (*n* = 856) had a mean IPWC of 1.13 kg/m^2^ (SD 3.5). 49.9% (*n* = 427) experienced a BMI increase (> 1 kg/m^2^). IPWC rates observed across categories: IPWL: 167 (19.5%); NC 262 (30.6%); IPWG: 427 (49.9%) (moderate IPWG: 235 [27.5%] and increased IPWG: 192 [22.4%]). IPWC varied by ethnicity, socioeconomic deprivation, age, and BMI in the index pregnancy; in a multiple regression model with these variables, increasing age and BMI in the index pregnancy were associated with lower IPWC.

**Discussion:**

22.4% of the cohort experienced IPWG at a level associated with adverse perinatal outcomes. Elevated rates of increased IPWG were observed in priority birthing populations, consistent with populations highlighted by the PMMRC. Further research is required to understand the impact of IPWC in AoNZ birthing populations.

## 1 Introduction

Overweight and obesity are global public health concerns linked to a range of poor health outcomes [1], the incidence of which continues to rise [2]. In 2022/23, 32.6% of adults were classified as obese in Aotearoa/New Zealand (AoNZ) [3]. People of Māori and Pacific ethnicities, disabled people, and people living in the most deprived neighbourhoods experience higher rates of obesity [3]. Similarly, obesity in pregnancy is also socioeconomically patterned, with the highest burden experienced by communities of lower socioeconomic backgrounds [4]. Current weight‐related screening in AoNZ during pregnancy is based on a one‐off body mass index (BMI) calculation as outlined in the National Guidelines for Consultation with Obstetric and Related Medical Services (Referral Guidelines) [5]. As per these guidelines, consultation with obstetric services is offered to pregnant people with a BMI between 35 and 49.9 kg/m^2^ in addition to routine primary maternity care; while full transfer of care and clinical responsibility to obstetric services is recommended for all pregnant people with a booking or pre‐pregnancy BMI of ≥ 50 kg/m^2^ [5].

Inter‐pregnancy weight change (IPWC) describes the weight change observed from one pregnancy to the next. Although there is no universal measurement of IPWC, common measurements of IPWC include change in body weight (kilograms or pounds), the percentage change in body weight (%), recorded changes in the BMI category and changes in BMI (kg/m^2^). Change in BMI is the most widely adopted measurement quantifying IPWC. Research demonstrates increased rates of pregnancy complications, including gestational diabetes mellitus (GDM), gestational hypertension, pre‐eclampsia, caesarean section (CS) birth, large for gestational age birthweight (LGA) and preterm birth (PTB) following interpregnancy weight gain (IPWG) of as little as 1 BMI unit (1 kg/m^2^) [6, 7, 8, 9, 10, 11]. Unlike overweight and obesity, IPWC is not captured as part of routine maternity screening or data collection. To date, no studies have explored IPWC in AoNZ birthing populations.

Maternity care in AoNZ is free to all New Zealand residents and spans primary to tertiary‐level care. While the midwifery‐led, continuity‐of‐care model of the AoNZ maternity system is often celebrated as the gold standard for maternity care of low‐risk women [12], health inequities across the maternity system persist. This was highlighted in the 16th annual report of the Perinatal and Maternal Mortality Review Committee (PMMRC) reporting on mortality and morbidity in 2021. This report again identified Māori, Pacific peoples, Indian populations, those aged under 20, and those living in areas of high deprivation as priority populations consistently experiencing worse clinical outcomes [13].

The primary objective of this study was to examine the distribution of IPWC in a birthing population in AoNZ. The secondary objective was to investigate IPWC across priority populations identified by the PMMRC.

## 2 Materials and Methods

This was a retrospective cohort study conducted in the MidCentral District, in AoNZ. We used an existing dataset, extracted from clinical records, to identify people who birthed two consecutive pregnancies in the region between January 2016 and December 2021. Cases were excluded from the study if either pregnancy was a multiple gestation or if there was missing BMI data recorded for either pregnancy.

## 3 Data Collection

Palmerston North Hospital audits labour and birth outcomes for all births in the MidCentral District. The maternal identifiers for all births between 2016 and 2021 were manually searched to identify those who gave birth twice over the study period. Audit data and clinical records were then used to identify those who met the criteria of having had their first two singleton pregnancies in the district.

Ethnicity is recorded as part of routine care. Ethnicity was prioritised following the Ministry of Health Ethnicity Data protocols: Māori, Pacific Peoples, Asian, Middle East/Latin American/African, and Other Ethnicity [14]. The most recently documented ethnicity was used if a person's prioritised ethnicity changed between pregnancies.

The NZ deprivation score index is a 1–10 area‐based scale of socioeconomic deprivation in AoNZ (decile 1 represents the least deprived areas while decile 10 represents areas with high levels of socioeconomic deprivation) [15]. Street address was used to assign deprivation scores. The most recently documented address was used if address changed between pregnancies.

BMI was calculated using weight (kg) and height (m) measurements documented in the clinical record

IPWC was calculated by subtracting the pre‐pregnancy BMI of the index [first] pregnancy from that of the second pregnancy.

Demographic data (maternal age, home address [to generate deprivation score], ethnicity, gravida and parity) was extracted from the existing dataset and, when required, completed by accessing clinical records.

### 3.1 Statistical Analysis

The primary outcome was IPWC, represented as a change in BMI (kg/m^2^). IPWC was treated as both a continuous and categorical variable. IPWC categories (Table 1) were adopted from existing IPWC literature [7, 8, 9, 16, 17].

**TABLE 1:** Interpregnancy weight change categories.

Interpregnancy weight loss (IPWL)	BMI reduction of > 1 kg/m^2^
No change (NC)	BMI change of −1 to 0.9 kg/m^2^
IPWG	
Moderate IPWG	BMI increase of 1–3 kg/m^2^
Increased IPWG	BMI increase of > 3 kg/m^2^

Frequencies, proportions and means were used to summarise the distribution of IPWC. An analysis of variance (ANOVA) was conducted to compare mean weight change across groups to determine statistical significance. Linear regression models were used to analyse the IPWC outcome as a continuous variable. Effects from unadjusted and adjusted models were estimated for each factor. The associations between demographic factors and the IPWC outcome as a categorical variable were analysed using Pearson's chi‐square test. A two‐sided Type‐I error of 0.05 indicated statistical significance.

Where data was missing in the dataset, clinical records were searched. The absence of BMI both in the dataset and electronic records resulted in the exclusion of the case from the study. Missing demographic information resulted in the removal of cases from relevant sub‐category analysis.

### 3.2 Ethics

Ethical approval was obtained through the University of Otago's low‐risk research pathway (Ethics Committee reference number: HD21/067) and the MidCentral District Health Board Ethics Committee (Research ID: 2021.10.008).

## 4 Results

**FIGURE 1:**
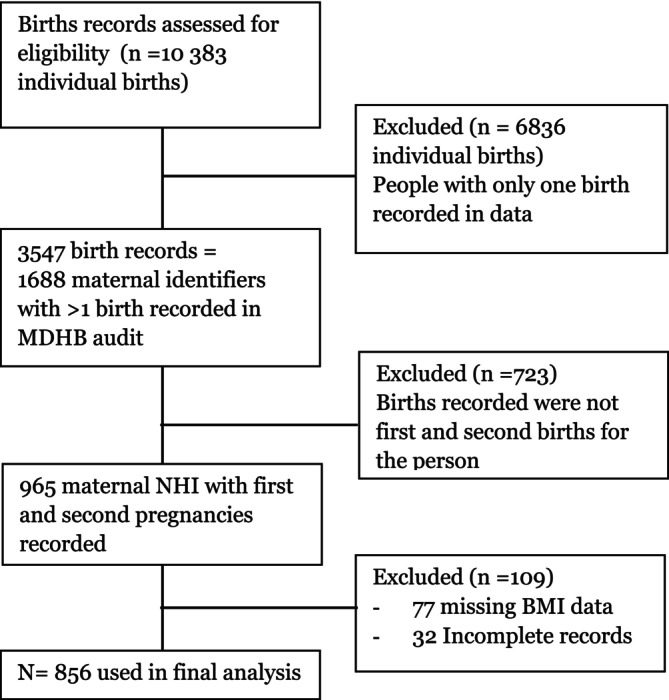
Cases included in final analysis.

Between January 2016 and December 2021, 10383 individual live births were recorded in the MidCentral District. Of these, 856 records were identified for inclusion in the final analysis (Figure 1). The mean age at delivery of the index pregnancy was 26.3 years (SD 5.0). The mean inter‐delivery interval was 27.5 months (SD 9.8) (Table 2).

**TABLE 2:** Demographic characteristics.

Study cohort		
Cohort size	*n* = 856	
Age at birth (index pregnancy)	26.3 (SD: 5.0)	
Inter delivery interval (months)	27.5 (SD: 9.8)	
Ethnicity		
Ethnicity group (prioritised)	*n*	%
European	583	68.1%
Māori	144	16.8%
Pacific	15	1.8%
Indian	27	3.2%
Other Asian	58	6.8%
Other	29	3.4%
Deprivation score		
Deprivation score	*n*	%
1–2 (least deprived)	77	9%
3–4	132	15.4%
5–6	183	21.4%
7–8	208	24.3%
9–10 (most deprived)	246	28.7%
Age group		
Age group (in years)	*n*	%
< 20	84	9.8%
20–24	235	27.5%
25–29	308	36.0%
30–34	179	20.9%
35–39	45	5.3%
> 40	3	0.4%
Index pregnancy BMI category		
BMI category	*n*	%
BMI < 18.5 underweight	13	1.5%
BMI 18.5–24.9 Normal weight	355	41.5%
BMI 25–29.9 Overweight	275	32.1%
BMI ≥ 30 Obese (total)	213	24.9
BMI 30–34.5 Obese I	118	13.8%
BMI 35–39.9 Obese II	57	6.7%
BMI ≥ 40 Obese III	38	4.4%

### 4.1 IPWC

The mean IPWC between the index and second pregnancy was +1.13 kg/m^2^ (SD 3.5) (Table 3). The study cohort consisted of 167 (19.5%) cases that experienced IPWL, 262 (30.6%) who maintained their BMI, 235 (27.5%) cases that experienced moderate IPWG, and 192 (22.4%) cases that experienced increased IPWG (Table 5).

**TABLE 3:** Mean interpregnancy weight change across the cohort and PMMRC priority populations.

	Full cohort		*p*
	Mean BMI change (SD)		
	BMI units (kg/m^2^)		
*n* = 856	1.13 (3.5)		
Ethnicity			
Ethnicity (prioritised)	Mean	SD	ANOVA
European	0.93	(3.36)	*p* = 0.193
Māori	1.60	(3.81)	
Pacific	1.65	(4.65)	
Indian	1.67	(1.92)	
Other Asian	1.75	(4.23)	
Other	0.88	(3.59)	
Deprivation score (NZDep2018)^a^			
Deprivation score	Mean	SD	ANOVA
1–2 (least deprived)	1.08	(2.29)	*p* = 0.492
3–4	1.32	(3.29)	
5–6	0.75	(3.38)	
7–8	1.33	(3.76)	
9–10 (most deprived)	1.24	(3.80)	
Age group			
Age (in years)	Mean	SD	ANOVA
< 20	1.67	(3.98)	***p* = < 0.05**
20–24	1.61	(4.06)	
25–29	0.89	(3.19)	
30–34	0.88	(2.72)	
35–39	0.59	(4.15)	
> 40	−1.77	(2.06)	
Index pregnancy BMI category			
BMI category	Mean	SD	ANOVA
BMI < 18.5 Underweight	1.68	(1.55)	***p* = < 0.001**
BMI 18.5–24.9 Normal weight	1.42	(2.46)	
BMI 25–29.9 Overweight	1.42	(3.31)	
BMI ≥ 30 Obese‐Total			
BMI 30–34.9 kg Obese (Class I)	0.75	(4.96)	
BMI 35–39.9 Obese (Class II)	−0.11	(3.57)	
BMI ≥ 40 Obese (Class III)	−0.78	(6.28)	

^a^ NZDep2018 is a 1 to 10 area‐based scale of socioeconomic deprivation in AoNZ displayed as deciles. Decile 1 represents areas with the least deprived scores; decile 10 represents areas with the most deprived scores.

### 4.2 Ethnicity

IPWC categories varied by ethnicity (*p* = < 0.05). Moderate IPWG was experienced by 12 (44.4%) of people identifying as Indian and 18 (31%) of those who fell into the ‘Other Asian’ ethnicity category. Increased IPWG was observed in 39 (27.1%) cases of people identifying as Māori compared to 119 (20.4%) of those identifying as NZ European. Conversely mean IPWC did not vary significantly across ethnicities (*p* = 0.193). Similarly, in a univariable model, Māori on average experienced 0.67 kg/m^2^ higher IPWC than NZ Europeans (95% CI: 0.03–1.31). However, there was no evidence of difference when adjusted for age, deprivation and BMI in the index pregnancy.

### 4.3 Deprivation Score

Increased IPWG occurred in 9 (11.7%) cases of birthing people living in areas with the least deprivation (deprivation scores 1 & 2). In those with higher rates of socioeconomic deprivation, the rate of increased IPWG was 22.4% (*n* = 41) to 25% (*n* = 33) (*p* = < 0.005). However, no evidence of differences in IPWC by deprivation was detected in a model adjusted for ethnicity, age and BMI in the index pregnancy.

### 4.4 Age Group

Mean BMI change decreased with increasing maternal age at the time of the first birth (*p* = < 0.05) in both unadjusted and adjusted models (Tables 3 and 4). Increased IPWC was more prevalent in younger birthing people (*p* = < 0.001): Of the 84 cases < 20 years, 29 (34.5%) experienced increased IPWG. Similarly, of the 235 cases aged 20 to 24 years, 72 (30.6%) experienced increased IPWG. Increased IPWG was observed in 56 (18.2%) aged 25–29 years; 26 (14.5%) aged 30–34; and 9 (20%) cases aged 35–39 at the time of the first birth (Table 5).

**TABLE 4:** Association between sub‐groups and IPWC.

	Unadjusted		Adjusted	
	Coef. Est b (95% CI)	*p*	Coef. Est b (95% CI)	*p*
Variables				
Ethnicity (reference: NZ European)				
Māori	**0.67 (0.03, 1.31)**	0.040*	0.49 (−0.18, 1.16)	0.154
Pacific	0.72 (−1.08, 2.52)	0.432	0.87 (−0.91, 2.65)	0.336
Indian	0.73 (−0.62, 2.09)	0.288	0.62 (−0.72, 1.96)	0.364
Other Asian	0.82 (−0.13, 1.76)	0.091	0.71 (−0.24, 1.65)	0.143
Other	−0.05 (−1.36, 1.25)	0.936	0.14 (−1.15, 1.44)	0.827
Deprivation (NZDep)	0.02 (−0.07, 0.11)	0.736	−0.01 (−0.11, 0.08)	0.767
Maternal age (in years)	**−0.08 (−0.13, −0.03)**	0.001*	**−0.08 (−0.13, −0.03)**	0.002*
BMI (1st pregnancy)	**−0.09 (−0.13, −0.05)**	< 0.001*	**−0.09 (−0.13, −0.05)**	< 0.001*

*Note:* Bolded results indicate significant findings at *p* < 0.05.

**TABLE 5:** IPWC observed in categories across study cohort and PMMRC priority populations.

	Study cohort								*p*
	Weight loss		No change		Moderate IPWG		Increased IPWG		
	(> − 1 kg/m^2^ change)		(−1 to 0.9 kg/m^2^ change)		(> 1 to 3 kg/m^2^ increase)		(> 3 kg/m^2^ increase)		
	*n*	%	*n*	%	*n*	%	*n*	%	
Study Cohort	167	19.5%	262	30.6%	235	27.5%	192	22.4%	
Ethnicity									
Ethnicity (prioritised)	Weight loss		No change		Moderate gain		Increased weight gain		Chi squared test
European	116	19.9%	189	32.4%	159	27.3%	119	20.4%	*p* = < 0.05
Māori	31	21.5%	31	21.5%	43	29.9%	39	27.1%	
Pacific	4	26.7%	3	20.0%	2	13.3%	6	40.0%	
Indian	1	3.7%	9	33.3%	12	44.4%	5	18.5%	
Other Asian	9	15.5%	17	29.3%	18	31.0%	14	24.1%	
Other	6	20.7%	13	44.8%	1	3.4%	9	31.0%	
Deprivation score (NZDep2018)^a^									
Deprivation Score	Weight loss		No change		Moderate gain		Increased weight gain		Chi squared test
1–2 (least deprived)	6	7.8%	42	54.5%	20	26.0%	9	11.7%	***p* = < 0.005**
3–4	23	17.4%	40	30.3%	36	27.3%	33	25.0%	
5–6	43	23.5%	52	28.4%	47	25.7%	41	22.4%	
7–8	40	19.2%	61	29.3%	59	28.4%	48	23.1%	
9–10 (most deprived)	53	21.5%	64	26.0%	68	27.6%	61	24.8%	
Age group									
Age (in years)	Weight loss		No change		Moderate gain		Increased weight gain		Chi Squared Test
< 20	22	26.2%	15	17.9%	18	21.4%	29	34.5%	***p* = < 0.001**
20–24	50	21.3%	56	23.8%	57	24.3%	72	30.6%	
25–29	57	18.5%	107	34.7%	88	28.6%	56	18.2%	
30–34	30	16.8%	64	35.8%	59	33.0%	26	14.5%	
35–39	6	13.3%	17	37.8%	13	28.9%	9	20.0%	
> 40	2	66.7%	1	33.3%	0	0.0%	0	0.0%	
Index pregnancy BMI category									
BMI category	Weight loss		No change		Moderate gain		Increased weight gain		Chi squared test
BMI < 18.5 Underweight	0	0.0%	6	46.2%	4	30.8%	3	23.1%	***p* = < 0.001**
BMI 18.5–24.9 Normal Weight	40	11.3%	134	37.7%	112	31.5%	69	19.4%	
BMI 25–29.9 Overweight	58	21.1%	69	25.1%	80	29.1%	68	24.7%	
BMI ≥ 30 Obese – Total	69	32%	53	24.9%	39	18.3%	52	24.4%	
BMI 30–34.9 kg Obese (Class I)	36	30.5%	29	24.6%	23	19.5%	30	25.4%	
BMI 35–39.9 Obese (Class II)	18	31.6%	17	29.8%	10	17.5%	12	21.1%	
BMI ≥ 40 Obese (Class III)	15	39.5%	7	18.4%	6	15.8%	10	26.3%	

*Note:* Bolded results indicate significant findings at *p* < 0.05.

^a^ NZDep2018 is a 1 to 10 area‐based scale of socioeconomic deprivation in AoNZ displayed as deciles. Decile 1 represents areas with the least deprived scores; decile 10 represents areas with the most deprived scores.

### 4.5 BMI Category

IPWC varied by BMI category in the index pregnancy (*p* = 0.001). Increasing index pregnancy BMI was associated with reduced IPWC: for every 1 kg/m^2^ increase associated with a 0.09 kg/m^2^ reduction in subsequent IPWC (Tables 3 and 4).

Nearly one‐third (*n* = 112, 31.5%) of cases with a normal BMI at the start of the index pregnancy experienced moderate IPWG and 69 (19.4%) experienced increased IPWG (Table 5).

## 5 Discussion

This retrospective cohort study examines IPWC across a birthing population (856 birthing people) in AoNZ. Our findings reveal a mean IPWC of 1.13 kg/m^2^ (SD 3.5), and a mean inter‐birth interval of 27.5 months (SD 9.8).

Almost half of the cohort (49.9%, *n* = 427) experienced IPWG (> 1 kg/m^2^), This is consistent with international literature, where IPWG of > 1 kg/m^2^ was observed in 36.8%–57.2% [9, 11, 18, 19, 20] of study populations. Notably, 22.4% of the cohort experienced increased IPWG. A 2020 meta‐analysis [21] revealed that at this level, IPWG is associated with higher rates of GDM (odds ratio [OR]: 2.21, 95% confidence interval [CI] 1.53–3.19); preeclampsia (OR: 1.77, 95% CI 1.53–2.04); gestational hypertension (OR: 1.78, 95% CI 1.61–1.97); CS (OR: 1.32, 95% CI 1.24–1.39). Consequently, nearly one‐quarter of birthing people in AoNZ enter their second pregnancy after experiencing IPWG, which may elevate their risk for these adverse outcomes.

IPWL was experienced by 19.5% of the cohort. Evidence examining IPWL and outcomes in subsequent pregnancies is less clear. While reduced rates of GDM [7, 22], gestational hypertension [9, 10, 21], CS birth [23], small for gestational age, and LGA [9, 10, 24] have been reported in the presence of IPWL, other studies report no significant change in rates of GDM [8, 9, 19, 25], gestational hypertension [9, 22], preeclampsia [9, 10, 23], CS [9, 19], PTB [9, 10, 25], SGA [25], and LGA [16, 24]. Some studies even note increased rates of SGA [24] and PTB [10, 24] following IPWL. Outcomes following IPWL likely depend on initial BMI and timing of weight loss in relation to subsequent pregnancies. In our cohort, one‐third of those with an obese BMI at baseline experienced IPWL. Since obesity represents a significant perinatal risk factor, it is hopeful that outcomes in this group will be more favourable following IPWL. Nonetheless, further research is needed to understand the significance of IPWL in birthing populations in AoNZ.

Among the cohort, 41.5% had a normal BMI at baseline, of those, 19.4% experienced increased IPWG between pregnancies. A 2019 meta‐analysis reported that people with a normal BMI at the start of their index pregnancy who went on to experience increased IWPG were more likely to experience complications in their second pregnancy than those with a baseline BMI > 25 kg/m^2^. Specifically, people with a BMI less than 25 kg/m^2^ who experienced IPWG of > 3 kg/m^2^ were more likely to develop GDM in their second pregnancy (OR 4.36, 95% CI 2.29–6.44) compared to those with a BMI over 25 kg/m^2^ (OR 2.2, 95% CI 1.40–3.12). Similar trends were noted for other outcomes, including pregnancy‐induced hypertension (BMI < 25 kg/m^2^: OR 2.21, 95% CI 1.18–2.60; BMI > 25 kg/m^2^: OR 1.37, 95% CI 1.16–1.59) and LGA birthweight (BMI < 25 kg/m^2^: OR 1.80 95% CI 1.24–2.35; BMI > 25 kg/m^2^: OR 1.50 95% CI 1.35–1.66) [26]. Currently, weight‐related screening practice in maternity care in AoNZ relies on pre‐pregnancy BMI, designating those with a BMI > 35 kg/m^2^ as at higher risk of developing pregnancy complications [5]. As such, individuals with a BMI < 25 kg/m^2^ in their first pregnancy who experience an increase of > 3 kg/m^2^ would not be identified as at increased risk of perinatal complications unless their BMI rose to > 35 kg/m^2^ before the second pregnancy. This gap in current screening practices may leave at‐risk individuals unmonitored and vulnerable to adverse outcomes. Future research should explore how BMI change, in addition to singular measurement, can enhance weight‐related screening in maternity care.

Our finding that almost 1 in 5 (19.4%) birthing people with a normal BMI will experience increased IPWG before their second pregnancy highlights a significant opportunity for timely lifestyle and weight management education. Postpartum guidelines in AoNZ do not make explicit recommendations for postpartum weight change; however, current primary healthcare guidelines recommend people with a BMI > 29.9 kg/m^2^ receive a holistic clinical examination and support to engage in weight management strategies [27]. The postpartum period is one of significant lifestyle, social and economic change for birthing people and their families. Our findings suggest that such education following birth should be universally incorporated into national policies for post‐partum care. Not limited to those currently experiencing overweight or obesity.

The secondary objective of this study was to investigate IPWC across priority populations identified by the PMMRC. Although this analysis was limited by small numbers within sub‐groups, variations between priority populations were observed across ethnicity, deprivation score and age.

Those < 20 years of age displayed the highest prevalence of increased IPWG (34.5%) relative to other age groups, and mean IPWC decreased with increasing maternal age at the time of the first birth (*p* = < 0.05) in both unadjusted and adjusted models. No differences in mean IPWC by ethnicity or deprivation score were observed in the adjusted model. In 2018, the median age of the Māori birthing population was younger (27) than that observed in the European population (30.8) [28]. It is likely that the observed effect of ethnicity (between Māori and NZ European cohorts) in the unadjusted model reflects a difference observed in the younger birthing cohort. Given the increased rate of adverse perinatal outcomes experienced by these groups, there remains a need for research examining IPWC within diverse, multi‐ethnic birthing populations in AoNZ.

Subgroup analyses utilised scales readily used in national datasets to improve the interpretation of results, such as BMI and NZDep. As such, limitations inherent to these scales can be applied to this study. Reliance on BMI as the primary measure of IPWC poses specific challenges when applied to an ethnically diverse population such as that found in AoNZ. Due to its Eurocentric origin, the use of BMI in ethnically diverse populations, has been questioned [29]. It has been suggested that the upper limit of normal‐range BMI ranges from < 22 to 25 kg/m^2^ in Asian populations [30] to < 26 kg/m^2^ for Māori and Pacific populations [29]. The correct interpretation of BMI in these populations is critical, given the overrepresentation of Māori, Pacific and Indian populations in adverse perinatal outcome data [13]. Therefore, the significance of IPWC in these populations may be under‐recognised, and as such, future research should focus on understanding IPWC within an ethnically diverse birthing population in AoNZ. NZDep is a small‐area measure of socio‐economic deprivation providing a proxy, or partial measure of a complex phenomenon. The index itself provides limited insight to the specific factors driving any variations in outcomes that may be observed between populations.

Strengths of this study include the utilisation of a dataset capturing all births in the study district. Data were extracted directly from clinical records, improving accuracy. Retrospective data collection and a small sample size limited the generalisability of findings and sub‐category analysis. Weight and height measurements entered to clinical records were self‐reported. Although self‐reported height and weight in pregnant populations has not been shown to bias associations between pregnancy‐related weight and birth outcomes [31], standardised height and weight measurements would have been preferable.

This is the first study examining IPWC in an AoNZ birthing population. Our findings highlight the need for continued research in this area. Future studies should examine the relationship between IPWC in AoNZ and the incidence of adverse perinatal outcomes. Moreover, understanding postpartum weight trajectory in the same birthing population should also be explored to contextualise IPWC research.

IPWG was experienced by almost half (49.9%) of this study cohort and varied by socioeconomic deprivation, ethnicity, age, and BMI categories. Current weight‐related screening practices are not sensitive to the potential increased risk posed by IPWC in AoNZ birthing populations. Our findings highlight the inter‐pregnancy period as a critical time to support healthy weight management in all birthing people, not limited to those currently experiencing overweight or obesity. Further research is necessary to understand how IPWC impacts pregnancy outcomes in AoNZ and should focus on understanding postpartum weight trajectory.

## Conflicts of Interest

The authors declare no conflicts of interest.

## References

[1] World Health Organization , Health Service Delivery Framework for Prevention and Management of Obesity (World Health Organisation, 2023), https://iris.who.int/bitstream/handle/10665/367784/9789240073234‐eng.pdf?sequence=1.

[2] World Health Organization Regional Office for Europe, World Health Organization. Regional Office for Europe , WHO European Regional Obesity: Report 2022 (World Health Organization, Regional Office for Europe, 2022).

[3] Ministry of Health , Annual Data Explorer 2022/23: New Zealand Health Survey [Data File] (Ministry of Health Manatū Hauora, 2023), https://minhealthnz.shinyapps.io/nz‐health‐survey‐2022‐23‐annual‐data‐explorer/.

[4] M. Marmot , Health Inequalities in the EU: Final Report of a Consortium (Publications Office of the European Union, 2014), https://op.europa.eu/en/publication‐detail/‐/publication/e3d84056‐2c24‐4bd3‐92db‐2cb71a0d0bc4/language‐en.

[5] A. Aratohu , K. Āwhina , T. Ratonga , et al., Guidelines for Consultation with Obstetric and Related Medical Services (Referral Guidelines) (Te Whatu Ora – Health New Zealand, 2023).

[6] A. Bogaerts , E. De Baetselier , L. Ameye , T. Dilles , B. Van Rompaey , and R. Devlieger , “Postpartum Weight Trajectories in Overweight and Lean Women,” Midwifery 49 (2017): 134–141. 27638342 10.1016/j.midw.2016.08.010

[7] S. F. Ehrlich , M. M. Hedderson , J. Feng , E. R. Davenport , E. P. Gunderson , and A. Ferrara , “Change in Body Mass Index Between Pregnancies and the Risk of Gestational Diabetes in a Second Pregnancy,” Obstetrics and Gynecology 117, no. 6 (2011): 1323–1330. 21606742 10.1097/AOG.0b013e31821aa358 PMC3222684

[8] L. M. Sorbye , R. Skjaerven , K. Klungsoyr , and N. H. Morken , “Gestational Diabetes Mellitus and Interpregnancy Weight Change: A Population‐Based Cohort Study,” PLoS Medicine 14, no. 8 (2017): e1002367. 28763446 10.1371/journal.pmed.1002367 PMC5538633

[9] E. Villamor and S. Cnattingius , “Interpregnancy Weight Change and Risk of Adverse Pregnancy Outcomes: A Population‐Based Study,” Lancet 368, no. 9542 (2006): 1164–1170. 17011943 10.1016/S0140-6736(06)69473-7

[10] J. M. Wallace , S. Bhattacharya , D. M. Campbell , and G. W. Horgan , “Inter‐Pregnancy Weight Change Impacts Placental Weight and Is Associated With the Risk of Adverse Pregnancy Outcomes in the Second Pregnancy,” BMC Pregnancy and Childbirth 14 (2014): 40. 24450357 10.1186/1471-2393-14-40 PMC3900734

[11] E. Villamor and S. Cnattingius , “Interpregnancy Weight Change and Risk of Preterm Delivery,” Obesity 24, no. 3 (2016): 727–734, 10.1002/oby.21384. 26833699

[12] P. Dawson , C. Jaye , R. Gauld , and J. Hay‐Smith , “Barriers to Equitable Maternal Health in Aotearoa New Zealand: An Integrative Review,” International Journal for Equity in Health 18, no. 1 (2019): 168. 31666134 10.1186/s12939-019-1070-7 PMC6822457

[13] Perinatal and Maternal Mortality Review Committee , Sixteenth Annual Report of the Perinatal and Maternal Mortality Review Committee | Te Pūrongo Ā‐Tau Tekau MĀ Ono o Te Komiti Arotake Mate Pēpi, Mate Whaea Hoki: Reporting Mortality and Morbidity 2021 | Te Tuku Pūrongo Mō Te Mate Me Te Whakamate 2021, vol. 2024 (Perinatal and Maternal Mortality Review Committee, 2024).

[14] Ministry of Health , HISO 10001:2017 Ethnicity Data Protocols (Ministry of Health, 2017).

[15] J. Atkinson , C. Salmond , and P. Crampton , NZDep201 Index of Deprivation (University of Otago, 2020), https://www.otago.ac.nz/__data/assets/pdf_file/0020/326711/nzdep2018‐index‐of‐deprivation‐research‐report‐final‐dec‐2020‐823833.pdf.

[16] N. Ziauddeen , S. Wilding , P. J. Roderick , N. S. Macklon , and N. A. Alwan , “Is Maternal Weight Gain Between Pregnancies Associated With Risk of Large‐For‐Gestational Age Birth? Analysis of a UK Population‐Based Cohort,” BMJ Open 9, no. 7 (2019): e026220, 10.1136/bmjopen-2018-026220. PMC6615839 31289065

[17] C. W. Ku , T. S. Cheng , C. O. Ku , et al., “Distribution and Association of Interpregnancy Weight Change With Subsequent Pregnancy Outcomes in Asian Women,” Research Square 13 (2022): 4834, 10.21203/rs.3.rs-1607168/v1. PMC10039003 36964283

[18] A. M. Dude , M. C. Smid , D. W. Branch , et al., “Interpregnancy Body Mass Index Change and Offspring Mortality Risk Following the Second Pregnancy,” American Journal of Perinatology 40, no. 4 (2023): 387–393. 33878768 10.1055/s-0041-1727230 PMC10552797

[19] C. R. Knight‐Agarwal , L. T. Williams , D. Davis , et al., “Association of BMI and Interpregnancy BMI Change With Birth Outcomes in an Australian Obstetric Population: A Retrospective Cohort Study,” BMJ Open 6, no. 5 (2016): e010667. 10.1136/bmjopen-2015-010667 PMC4874127 27165646

[20] N. Ziauddeen , J. Y. Huang , E. Taylor , P. J. Roderick , K. M. Godfrey , and N. A. Alwan , “Interpregnancy Weight Gain and Childhood Obesity: Analysis of a UK Population‐Based Cohort,” International Journal of Obesity 46, no. 1 (2022): 211–219, 10.1038/s41366-021-00979-z. 34645936 PMC8748200

[21] Y. E. G. Timmermans , K. D. G. van de Kant , E. O. Oosterman , et al., “The Impact of Interpregnancy Weight Change on Perinatal Outcomes in Women and Their Children: A Systematic Review and Meta‐Analysis,” Obesity Reviews 21, no. 3 (2020): e12974. 31751496 10.1111/obr.12974 PMC7050512

[22] A. R. Kruse , M. S. Darling , M. K. L. Hansen , M. J. Markman , F. F. Lauszus , and H. B. Wielandt , “Recurrence of Gestational Diabetes in Primiparous Women,” Acta Obstetricia et Gynecologica Scandinavica 94, no. 12 (2015): 1367–1372. 26342157 10.1111/aogs.12764

[23] A. M. Dude , A. D. Lane‐Cordova , and W. A. Grobman , “Interdelivery Weight Gain and Risk of Cesarean Delivery Following a Prior Vaginal Delivery,” American Journal of Obstetrics and Gynecology 217, no. 3 (2017): 373.e1–373.e6. 10.1016/j.ajog.2017.05.024 PMC5581260 28526451

[24] R. H. Benjamin , S. Littlejohn , M. A. Canfield , M. K. Ethen , F. Hua , and L. E. Mitchell , “Interpregnancy Change in Body Mass Index and Infant Outcomes in Texas: A Population‐Based Study,” BMC Pregnancy and Childbirth 19, no. 1 (2019): 119, 10.1186/s12884-019-2265-z. 30953457 PMC6451298

[25] R. D. McBain , G. A. Dekker , V. L. Clifton , B. W. Mol , and L. E. Grzeskowiak , “Impact of Inter‐Pregnancy BMI Change on Perinatal Outcomes: A Retrospective Cohort Study,” European Journal of Obstetrics, Gynecology, and Reproductive Biology 205 (2016): 98–104, 10.1016/j.ejogrb.2016.07.487. 27567535

[26] N. E. W. D. Teulings , K. L. Masconi , S. E. Ozanne , C. E. Aiken , and A. M. Wood , “Effect of Interpregnancy Weight Change on Perinatal Outcomes: Systematic Review and Meta‐Analysis,” BMC Pregnancy and Childbirth 19, no. 1 (2019): 386. 31660893 10.1186/s12884-019-2566-2 PMC6819632

[27] Ministry of Health , Clinical Guidelines for Weight Management in New Zealand Adults (Ministry of Health, 2017), https://www.health.govt.nz/system/files/documents/publications/clinical‐guidelines‐for‐weight‐management‐in‐new‐zealand‐adultsv2.pdf.

[28] Statistics New Zealand , Parenting and Fertility Trends in New Zealand: 2018 (Statistics New Zealand, 2019), https://www.stats.govt.nz/reports/parenting‐and‐fertility‐trends‐in‐new‐zealand‐2018/.

[29] G. Sundborn , P. A. Metcalf , D. Gentles , et al., “Overweight and Obesity Prevalence Among Adult Pacific Peoples and Europeans in the Diabetes Heart and Health Study (DHAHS) 2002‐2003, Auckland New Zealand,” New Zealand Medical Journal 123, no. 1311 (2010): 30–42. 20360794

[30] WHO Expert Consultation , “Appropriate Body‐Mass Index for Asian Populations and Its Implications for Policy and Intervention Strategies,” Lancet 363, no. 9403 (2004): 157–163, 10.1016/S0140-6736(03)15268-3. 14726171

[31] I. Headen , A. K. Cohen , M. Mujahid , and B. Abrams , “The Accuracy of Self‐Reported Pregnancy‐Related Weight: A Systematic Review,” Obesity Reviews 18, no. 3 (2017): 350–369. 28170169 10.1111/obr.12486

